# How Do Plant Growth-Promoting Bacteria Use Plant Hormones to Regulate Stress Reactions?

**DOI:** 10.3390/plants13172371

**Published:** 2024-08-26

**Authors:** Anna M. Timofeeva, Maria R. Galyamova, Sergey E. Sedykh

**Affiliations:** 1 SB RAS Institute of Chemical Biology and Fundamental Medicine, 630090 Novosibirsk, Russia; anna.m.timofeeva@gmail.com; 2 Faculty of Natural Sciences, Novosibirsk State University, 630090 Novosibirsk, Russia; mgalyamova@gmail.com

**Keywords:** PGPB, phytohormones, plant growth stimulation, auxin, abscisic acid, cytokinins, ethylene, plant hormones, salicylic acid, deaminase, biofertilizers

## Abstract

Phytohormones play a crucial role in regulating growth, productivity, and development while also aiding in the response to diverse environmental changes, encompassing both biotic and abiotic factors. Phytohormone levels in soil and plant tissues are influenced by specific soil bacteria, leading to direct effects on plant growth, development, and stress tolerance. Specific plant growth-promoting bacteria can either synthesize or degrade specific plant phytohormones. Moreover, a wide range of volatile organic compounds synthesized by plant growth-promoting bacteria have been found to influence the expression of phytohormones. Bacteria–plant interactions become more significant under conditions of abiotic stress such as saline soils, drought, and heavy metal pollution. Phytohormones function in a synergistic or antagonistic manner rather than in isolation. The study of plant growth-promoting bacteria involves a range of approaches, such as identifying singular substances or hormones, comparing mutant and non-mutant bacterial strains, screening for individual gene presence, and utilizing omics approaches for analysis. Each approach uncovers the concealed aspects concerning the effects of plant growth-promoting bacteria on plants. Publications that prioritize the comprehensive examination of the private aspects of PGPB and cultivated plant interactions are of utmost significance and crucial for advancing the practical application of microbial biofertilizers. This review explores the potential of PGPB–plant interactions in promoting sustainable agriculture. We summarize the interactions, focusing on the mechanisms through which plant growth-promoting bacteria have a beneficial effect on plant growth and development via phytohormones, with particular emphasis on detecting the synthesis of phytohormones by plant growth-promoting bacteria.

## 1. Introduction

Sustainable agriculture is under threat because of a range of critical challenges, namely, floods, droughts, extreme temperatures, soil salinization, nutrient deficiencies, and the accumulation of toxic substances and pesticides resulting from human activity. These factors have an adverse impact on the growth of plants, resulting in a decrease in both the quantity and quality of food crops. It is for this reason that finding eco-friendly approaches for plant growth stimulation is crucial. Mineral fertilizers, mainly nitrogen, phosphorus, and potassium, are commonly used to achieve high yields in agriculture and horticulture [1,2]. However, there are concerns about their negative effects on the environment and human health [3,4]. The efficiency of mineral fertilizer use is not high; for example, the efficiency of nitrogen use by crops is less than 40%, and for wheat, it is as low as 31% [5], which means that the rest of the fertilizer is lost from agroecosystems through run-off and leaching, as well as volatilization and denitrification. In addition, the continued use of synthetic mineral fertilizers represents a significant financial burden for farmers as the cost of fertilizers continues to rise [6]. The search for innovative and sustainable methods to manage soil fertility and increase crop yields is urgent. One such approach is using plant growth-promoting bacteria (PGPB), a trend that is undoubtedly underway, given the numerous publications in scientific journals [7]. The use of PGPB as biofertilizers has great potential. PGPB improve plant nutrient uptake and protect crops from pests, diseases, and various types of stress [8]. Currently, various PGPB strains are employed as biofertilizers, and this number is expected to rise as additional PGPB strains are discovered and their functions are evaluated.

PGPB can directly stimulate the proliferation of their host plants in various ways, such as fixing atmospheric nitrogen and supplying it to plants [7], secreting iron-binding siderophores to extract soluble iron from the soil and supplying it to plants, thereby depriving fungal pathogens in the vicinity of soluble iron [9], and dissolving minerals and mineralizing organic compounds containing, for example, phosphorus [10]. Furthermore, they can produce low-molecular-weight metabolites (such as hydrogen cyanide) that have antifungal activity [11] or enzymes, such as protease, lipase, chitinase, or β-1,3-glucanase, capable of lysing some fungal cells [12], as well as displace phytopathogens from their ecological niche on the root surface [13]. Finally, PGPB can synthesize various phytohormones, and the mechanism of their synthesis is also a focus of this review.

**Figure 1:**
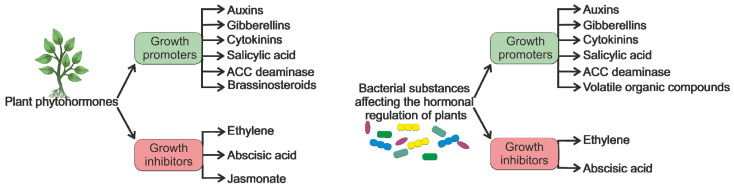
Classification of plant phytohormones and bacterial substances based on their impact on plant growth.

The physiological functions of plants are regulated by a wide range of phytohormones including abscisic acid, salicylic acid, cytokinins, gibberellins, brassinosteroids, auxins, jasmonic acid, ethylene, and numerous others. Phytohormones do not function independently; they interact cooperatively or in opposition [14]. Phytohormones can be classified into two groups based on their impact on plants as follows: growth-promoting and growth-inhibiting hormones (Figure 1).

The presence of bacteria in the soil and plant tissues can alter the levels of phytohormones, leading to changes in plant growth, development, and the ability to cope with stress. Numerous plant growth-promoting bacteria have been found to possess the capability of synthesizing or degrading certain plant phytohormones [15]. Furthermore, PGPB can produce several volatile organic compounds (VOCs) [16], directly enhancing plant growth or indirectly impacting phytohormone expression.

Phytohormones play a crucial role in enabling plants to adapt to both biotic and abiotic environmental factors. They regulate the expression of plant genes, facilitating a plant’s response to fluctuations in its surroundings. Moreover, it is important to highlight that plants can exploit the intrinsic phytohormone degradation activity exhibited by various soil bacteria to uphold optimal levels of different phytohormones [17].

The main objective of this review is to assess how PGPB influence plant growth and development, particularly in terms of hormonal regulation and their response to biotic and abiotic stress factors. In the following sections, we will delve into the effects of microbial phytohormones on plant growth and development, the interplay among different phytohormones, and techniques for identifying the synthesis of phytohormones by PGPB.

## 2. PGPB and Phytohormones in the Rhizosphere

The impact of PGPB on plants can vary depending on the prevailing growth conditions. In optimal conditions, such as when the soil is abundant in nutrients, PGPB frequently do not manifest a noticeable impact on the growth and development of plants [18,19]. Nevertheless, the prominence of bacterial–plant interactions becomes more apparent when faced with abiotic stressors such as salinization, drought, heavy metal pollution, and insufficient mineral nutrients.

The secretion of cytokinins, auxins, gibberellins, and other phytohormones by specific bacteria has been observed to increase plant growth and mitigate the toxic effects of metals [20]. The specific type of phytohormones dictates their crucial role in initiating seed germination, promoting root and shoot growth, and facilitating leaf expansion. These effects are achieved through mechanisms such as cell elongation, division, and differentiation and are complemented by other positive impacts. A study was conducted to evaluate the impact of water deficit conditions on phytohormone content in potato microplants. The findings demonstrated that inoculation with *Azospirillum brasilense* or *Ochrobactrum cytisi* resulted in enhanced leaf mass accumulation. This effect was attributed to the elevated content of phytohormones in the plant stems and leaves [21].

Among the auxins synthesized by bacteria, indole-3-acetic acid (IAA) holds the utmost significance. Studies have found that IAAs are manufactured by approximately 80% of the bacteria inhabiting the rhizosphere, including those that form relationships with plants. This suggests that the effects on plant growth are largely influenced by IAA-related processes [22]. Furthermore, it is known that microorganisms can produce the enzyme 1-aminocyclopropane-1-carboxylate (ACC) deaminase, which serves as a regulator for ethylene concentrations in plants [23]. When exposed to unfavorable conditions, plants produce ethylene, a plant stress hormone that can have harmful effects when its concentration becomes excessive. Ethylene is widely recognized as a multifunctional plant hormone responsible for regulating both growth and aging [24]. Consequently, the presence of ACC deaminase is crucial for maintaining the balance of this phytohormone [25].

PGPB can alter the concentration of phytohormones in the rhizosphere, such as auxins [26] and ethylene [27], by utilizing the hormones or their precursors as carbon and/or nitrogen sources. It was found, for example, that *Serratia proteamaculans* can use the cytokinin N6-benzyladenine as a sole carbon source [28], and *Azospirillum* can produce gibberellins [29]. Several *Pseudomonas* strains were reported to catabolize salicylic acid [30,31]. *Achromobacter*, *Burkholderia*, and *Pseudomonas* strains are known to degrade indole-3-acetic acid and salicylic acid [32]. The application of *Azospirillum* was found to result in an elevation in corn root volume, plant height, and shoot count in wheat. The authors of that study suggested that the impact on wheat could be ascribed to the phytohormones generated by the bacteria [33]. Although *Azospirillum* has been widely reported to have the N_2_ binding ability, a growing number of studies describe its involvement in stimulating plant growth. One of the main properties of *Azospirillum* is based on the synthesis of phytohormones and other compounds, including gibberellins, abscisic acid, auxins, cytokinins, ethylene, and salicylic acid [34,35,36].

The following subsections consider the specifics of different phytohormones.

### 2.1. Auxins

**Figure 2:**
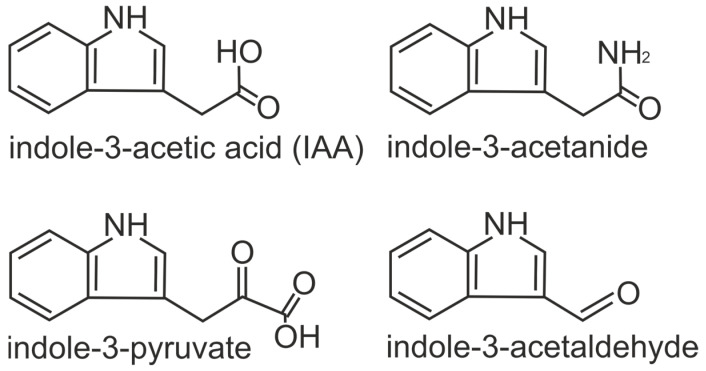
Auxin (indole-3-acetic acid) and its derivatives.

The first phytohormone to be identified was auxin, also known as indole-3-acetic acid. Various physiological processes rely heavily on the function of auxin, which serves as a regulator for plant growth and development [37]. Among the various biologically active auxins, the focus of the scientific literature is primarily on IAA [38]. Compounds such as indole-3-pyruvate, indole-3-acetamide, and indole-3-acetaldehyde exhibit auxin-like activity (Figure 2). Examples of inactive auxin forms include 4-chloroindole-3-acetic acid and others that can form complexes with alcohols, amino acids, sugars, and glycoproteins [39].

The main precursor for IAA synthesis is the amino acid tryptophan. When added to a culture medium, tryptophan enhances the production of IAA by bacteria. At least five pathways have been identified and described for the synthesis of IAA from tryptophan, with most of these pathways resembling those observed in plants, although with some variations in intermediates [40,41]. For example, several IAA production pathways have been demonstrated for the genus *Azospirillum* [38,42]. A comprehensive analysis of the regulation and pathways of auxin biosynthesis in bacteria is provided in the review [43]. The rationale behind the existence of multiple IAA biosynthesis pathways may be that detrimental mutations in one pathway do not interfere with the operation of another biosynthesis pathway. As a result, they do not reduce the effectiveness of the PGPB strain in facilitating plant growth and development. It has been demonstrated that *Burkholderia phytofirmans* [44], *Pseudomonas putida* [45], *Methylobacterium* [46,47], and bacteria of the genus *Rhizobium* [48] can degrade IAA.

Auxin serves as a signaling molecule for various groups of microorganisms [43] and can therefore have a major impact on microbial–plant interactions. The interference of microorganisms with plant development is well documented, particularly in the case of phytopathogenic bacteria like *Agrobacterium* and *Pseudomonas savastanoi*. These bacteria induce tumors and galls by disrupting the balance of auxin in plants [49]. In contrast, plant growth-promoting rhizobacteria, specifically *Azospirillum sobobobacterium*, can induce plant root development by synthesizing auxin [38]. The inoculation of PGPB plants induces changes in root architecture, primarily characterized by an increase in root hairs and lateral roots, as well as a reduction in root length. The expansion of the root surface area can lead to the generation of more root exudates, which function as a substrate for the development of microbial communities in the rhizosphere [50,51].

It is assumed that up to 80% of bacteria isolated from the rhizosphere of higher plants can synthesize IAA [40,41]. IAA products have been reported in various PGPB genera, such as *Acetobacter*, *Acinetobacter*, *Azospirillum*, *Arthrobacter*, *Azotobacter*, *Bacillus*, *Bradyrhizobium*, *Burkholderia*, *Herbaspirillum*, *Klebsiella*, *Mesorhizobium*, *Paenibacillus*, *Pantoea*, *Pseudomonas*, *Rhizobium*, *Rhodococcus*, *Serratia*, *Strenotophomonas*, *Streptomyces*, and *Rouxiella* [52,53].

To construct a PGPB overproducing IAA, the construct p-iaaMtms2 was introduced into the *Sinorhizobia meliloti* (RD64) strain [54,55,56]. Using a transformed strain of *S. meliloti* for *Medicago truncatula* nodulation resulted in higher tolerance to high salt (0.3 M) and several other stresses.

Similar results were obtained for a genetically modified strain of *Azospirillum brasilense*, in which the auxin biosynthesis gene *ipdC* was placed under the control of either the constitutive promoter PnptII or the root exudate responsive promoter PsbpA. This genetic modification enhanced IAA biosynthesis, and inoculation with this strain causes an increase in wheat shoot biomass [57].

The role of mutualism between algae and PGPB in the regulation of environmental IAA levels has been reported. Such interactions have been shown for *Chlamydomonas reinhardtii* [58] and PGPB of the genus *Methylobacterium* [47,59]. The use of L-amino acids and short peptides as a carbon source by *Chlamydomonas* algae has been reported via L-amino acid oxidase (LAO1) [46]. LAO1 oxidizes L-amino acids to produce ammonium, hydrogen peroxide, and keto acids [60]. Some of these keto acids, e.g., indolepyruvic acid and phenylpyruvic acid, are known precursors in auxin biosynthesis [61,62], and their inoculation leads to the accumulation of auxin in the environment. High levels of IAA inhibit algal cell proliferation, and these inhibitory effects can be alleviated by the presence of the plant growth-promoting bacterium *Methylobacterium aquaticum* [46], the growth of which is synergistic with the presence of the algae. *M. aquaticum* is thought to play a role in IAA degradation.

### 2.2. Cytokinins

Cytokinins (CKs), which are endogenous plant hormones, play a crucial role in multiple physiological processes at low concentrations. These processes include cell division, organ formation, shoot and root development, accessory bud growth, chlorophyll biosynthesis, and nutrient transport, among others [63,64]. The prevalent type of CK found in plants is zeatin, which was first discovered in *Zea mays* (corn) during the 1950s [65].

**Figure 3:**
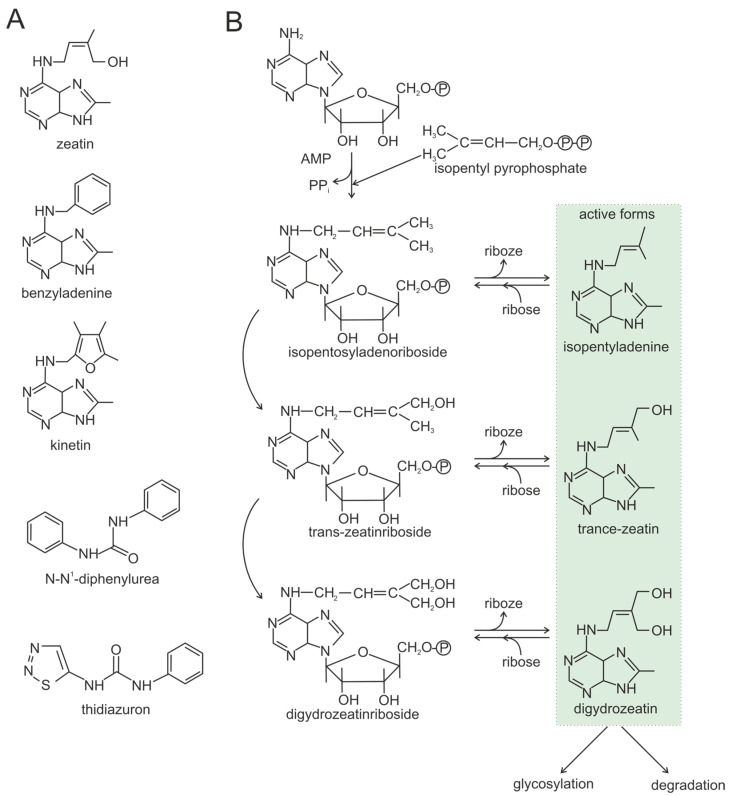
(**A**). Chemical structures of some cytokinins. (**B**) Example of the biosynthesis pathway of some cytokinins. The active forms of cytokinins are indicated in green.

Several types of CKs are classified, and some of them are shown in Figure 3A [66]. depending on the configuration of the substituent at the N6-adenine position–isoprenoid and/or aromatic groups, CKs have significantly different biochemical properties, receptor affinity [67,68], biological activities, and abundances in plants [69,70,71]. The predominant natural isoprenoid CKs are N6-(Δ2-isopentenyl)adenine, trans-zeatin, cis-zeatin, and dihydroseatin (DHZ) [72].

The inclusion of ribose in the CK skeleton results in a form with weak activity [73], while the inclusion of glucose produces reversible storage and irreversible deactivation forms known as O- and N-glucosides, respectively [74,75]. The action of numerous CK representatives allows them to be classified into two primary categories. Some CKs, such as trans-zeatin and isopentenyladenine, exhibit a potent yet short-lived effect, while others, such as cis-zeatin, operate through a more subtle and sustained mechanism [76].

CK biosynthesis is initiated by the enzyme isopentenyltransferase (IPT) encoded by the *ipt* gene. PGPB can produce CKs by expressing IPT, which is the key rate-limiting enzyme controlling CK production [77,78]. This gene was first detected in the soil bacterium *Agrobacterium tumefaciens* [79]. Homologues of the *ipt* gene have also been found in other PGPB genomes including *Pseudomonas psychrotolerans* [80] and *Nostoc* sp. [81]. However, it was later shown that *ipt* genes are rare among bacteria. Most bacterial species containing the *ipt* gene have been classified as either plant pathogens or PGPB, while a characteristic feature of pathogenic bacteria is the duplication of the *ipt* gene. Moreover, genomic neighborhood analysis showed that pathogen-associated *ipt* genes tended to cluster with other pathogen-associated genes. In contrast, PGPB-related *ipt* genes clustered with genes of other enzymes related to the CK biosynthetic pathway [78]. This gene clustering may be related to the function of common pathogen strain virulence pathways and PGPB–plant pathways through co-expression with other genes in the cluster.

Within the cellular environment, CKs are derived from adenine. The CK skeleton is formed when adenine is attached to a lateral isopentyl group, followed by the sequential removal of the phosphate group and ribose. Other CKs are formed by modification of the isopentyl moiety, including hydroxylation, oxidation, and reduction (Figure 3B).

CKs exert diverse effects on various types of plant cells. They can modulate cell division, seed germination, xylem and chloroplast differentiation, apical dominance, root elongation, transition to reproductive growth phase, development of flowers and fruits, leaf senescence, nutritional signaling, and pathogen–plant interactions [82,83,84,85]. One transcriptome analysis revealed over 100 CK-sensitive genes linked to photosynthesis, chlorophyll biosynthesis, and plastid gene expression in *Arabidopsis thaliana* [86]. These genes have been found to have a direct or indirect impact on various plant physiological processes, such as regulating germination of seeds, shoot proliferation and elongation, inducing flowering, fruiting and seed setting, and promoting senescence [87].

A number of bacteria were demonstrated to be capable of synthesizing CKs. These include *Methylobacterium*, *Bacillus licheniformis*, *Pseudomonas fluorescens*, *Bradyrhizobium japonicum*, and *Pseudomonas putida* [88,89]. Certain types of bacteria can produce the following CKs: zeatin, zeatin riboside, and isopentenyladenine [90]. The expression of CK genes by PGPB has the potential to significantly modify the phytohormonal composition of inoculated plants. For example, when lettuce was inoculated with *Bacillus subtilis* bacteria, the increase in CKs resulted in increased plant growth [91].

A genetically engineered strain of *Sinorhizobium meliloti* LMG202 carrying the zeatin biosynthesis gene *ipt* under the control of the lac promoter [92] was characterized by CK overproduction and tested for the potential to protect alfalfa plants under drought conditions [92]. This strain exhibited a five-fold increase in CK production compared with the wild-type bacteria. Following an extended period of severe drought, a significant enlargement in size was observed in the alfalfa plants that had been inoculated with the transformed strain compared with the plants that had been inoculated with the untransformed strain. CK production was evaluated in 46 bacterial strains of the genus *Methylobacterium*, as stated in [93]. Most of these strains are characterized by a high level of CK production, including trans-zeatin—the most active form of cytokinins.

*Pseudomonas* have also been reported to produce CKs [94]. For example, inoculation of tomato plants with CK-producing *Pseudomonas fluorescens* strain G20-18 was observed to enhance plant growth and drought tolerance.

### 2.3. Gibberellins

Gibberellic acids (GAs) modulate various developmental processes in plants [95], including germination, growth processes, stem elongation, seed germination, flowering, and fruit set [96]. Additionally, they are known to increase photosynthetic efficiency and chlorophyll content [97,98].

**Figure 4:**
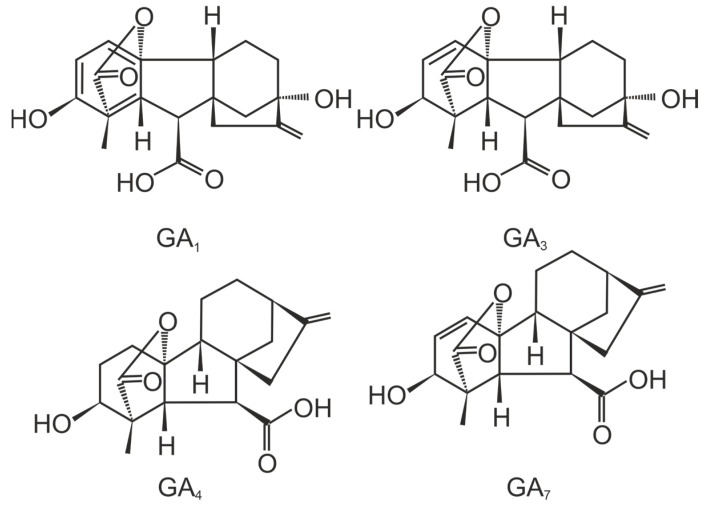
Chemical structure of some gibberellic acids.

Gibberellins encompass a wide range of tetracyclic diterpenoid carboxylic acids with either a C20 or C19 carbon skeleton (Figure 4). Over 130 distinct types of GAs have been identified and categorized as GA 1 to GA 136 [99,100]. A limited number of gibberellic acids have been identified in bacteria, specifically GA 1, GA 3, GA 4, and GA 20 [101]. It is worth noting that GA 1 and GA 4 exhibit the highest level of activity [102], and it should be emphasized that the effects of various gibberellins on plant tissues differ significantly. The most prevalent variant of this phytohormone is gibberellin GA3, with commercially purified versions of this compound widely accessible. Other (inactive) gibberellins are involved in the degradation of active gibberellins or their biosynthesis [103].

Several studies have shown the ability of *Streptomyces laurentii*, *Sinorhizobium*, *Bacillus safensis*, and *Bradyrhizobium* to secrete GA [89], causing these PGPBs to significantly improve the growth of bean plants and *Abelmoschus esculentus* [88]. The production of GA has been described for the following bacteria and genera: *Achromobacter xylosoxydans*, *Gluconobacter dizotropicus*, *Acinetobacter calcoaceticus*, *Rhizobia*, *Azotobacter*, *Bacillus*, *Herbaspirillum seropedicae*, and *Azospirillum* [27].

### 2.4. Salicylic Acid

The application of salicylic acid (SA) in plants has been found to confer systemic tolerance and mitigate various abiotic stresses such as extreme temperatures, salinity, heavy metal contamination, low oxygen levels, exposure to toxic organic compounds, ultraviolet radiation, and drought [104]. SA has been reported to stimulate flowering, ion uptake, nutrient transport, plant stomata movement, and protein biosynthesis [105]. SA can bind to specific amino acids, such as proline and arginine, and consequently increase a plant’s ability to withstand various environmental stressors.

Research has demonstrated the synergistic action of SA and PGPB when used together. For example, one study demonstrated that the addition of salicylic acid, along with PGPB, to chickpea plants facing severe salt stress substantially augmented the ameliorative effect of PGPB [106]. Another scientific investigation demonstrated that the growth of white bean plants can be improved under drought stress by co-inoculating them with two PGPB strains (*Bacillus subtilis* and *Pseudomonas putida*) and treating them with salicylic acid. The plants treated with SA showed superior growth compared with the untreated ones [107]. One other study discovered that the combined application of SA and PGPB (*Bradyrhizobium* sp. strain W100) favorably affected the growth of vigna plants in drought conditions [108].

SA biosynthesis in plants occurs via the isochorismate synthase (the main one) and the phenylalanine ammonia-lyase pathways [109,110,111]. Both pathways start with chorismate in plant plastids and differ among plant species. In bacteria, SA is synthesized as a secondary metabolite closely related to other pathways (e.g., siderophore synthesis) [112,113]. Typically, such metabolites are encoded by a biosynthetic gene cluster, which typically encodes all the enzymes required for the synthesis of the secondary metabolite [114]. Two general biosynthetic gene cluster systems, i.e., non-ribosomal peptide synthetases and polyketide synthases, are involved in most secondary metabolite synthesis processes [115]. *Pseudomonas*, *Bacillus, Azospirillum*, *Salmonella*, *Achromobacter*, *Vibrio*, *Yersinia*, and *Mycobacteria* have been reported to synthesize salicylates through these biosynthetic gene clusters. Additionally, both plant and bacterial SA can undergo various modifications to serve their distinct purposes [114]. Bacterial salicylate biosynthesis is often associated with the biosynthesis of catecholate-type siderophores [9]. Bacterial synthesis of SA acts as a precursor for siderophores, facilitating bacterial growth in the absence of iron [9].

### 2.5. Abscisic Acid

Abscisic acid (ABA) regulates numerous plant life cycle processes [116]. It modulates numerous plant physiological processes such as stress tolerance, senescence, and bud rest [117,118].

The balance between biosynthesis and catabolism regulates the ABA content in plant tissues. ABA is not deeply degraded during plant catabolism but transformed into inactive forms because of oxidation or conjugation reactions [119,120]. The constant breakdown of detached shoot tissues and root turnover results in the continuous delivery of substantial amounts of ABA and its catabolic products to the soil. According to the study by [121], the ABA transporters present in root epidermal cells can scavenge ABA, causing its concentration in the soil solution to rise progressively during the growing season.

**Figure 5:**
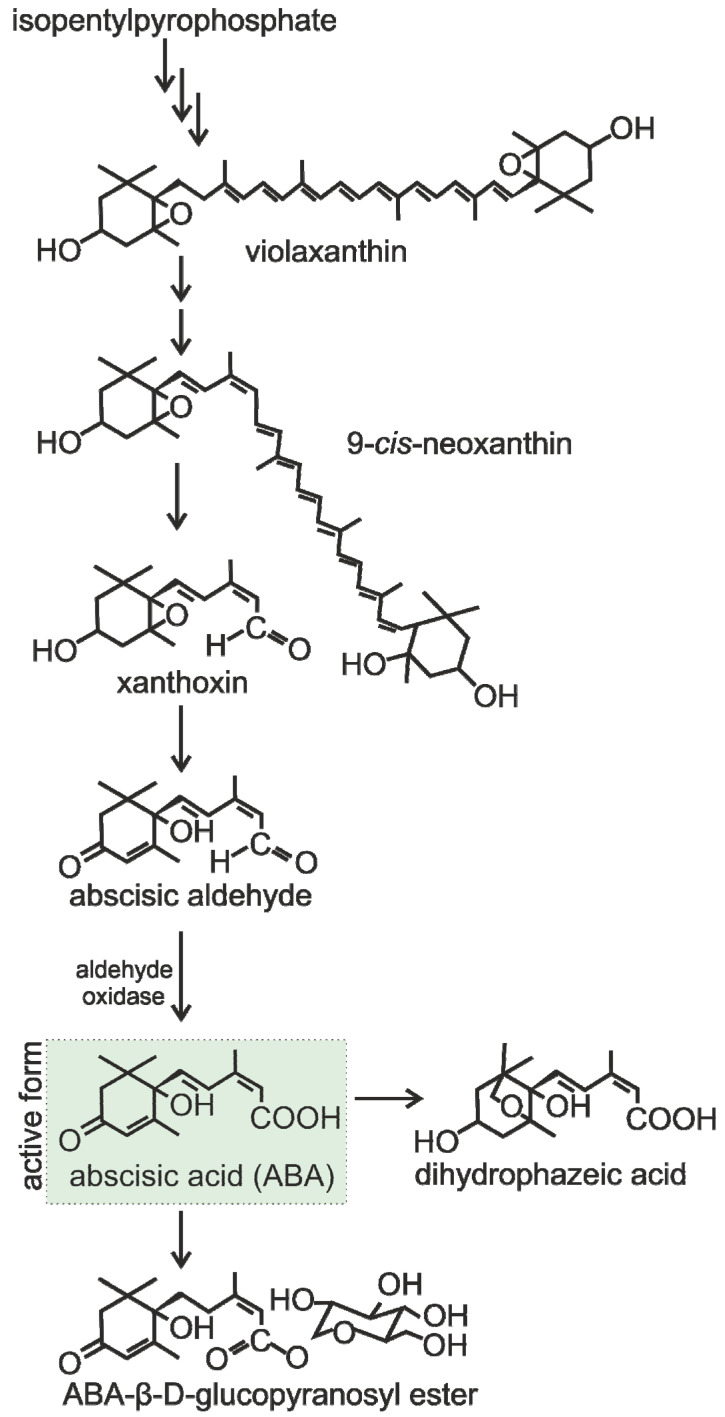
Synthesis and metabolism of ABA. The active form is shown in green.

Until recently, bacteria were thought not to synthesize ABA. However, in 2007, ABA was detected in cultures of endophytic bacteria in *Helianthus annuus* roots [122]. Then, the ability to synthesize ABA was found in several plant growth-promoting rhizobacteria, including *Azospirillum lipoferum* [123], *Arthrobacter koreensis* [123], *Achromobacter xylosoxydans*, *Bacillus licheniformis*, *Bacillus pumilus*, and *Brevibacterium halotolerans* [124]. The ABA biosynthesis pathway is closely associated with the violaxanthin cycle. The conversion of zeaxanthin to violaxanthin occurs, followed by its isomerization and cleavage into two unequal fragments—C15 (xanthoxin) and C25. The C25 fragment undergoes rapid degradation, and xanthoxin undergoes conversion into abscisic aldehyde, which is subsequently converted into ABA (Figure 5). Moreover, ABA has been identified at low concentrations in diverse living organisms, encompassing bacteria, cyanobacteria, algae, mosses, fungi, and lichens [119,125,126].

Two strains of *Rhodococcus* P1Y and *Novosphingobium* P6W were described [127], which can utilize ABA as a sole carbon source and reduce its concentration in tomato roots or leaves. The correlation observed between the impact of these bacteria on plant growth and the ABA concentration in plants implies that ABA-metabolizing rhizobacteria may engage with plants via an ABA-dependent mechanism. In other studies, maize plants treated with fluridone, an inhibitor of ABA synthesis, exhibited a growth inhibition similar to that caused by drought stress [90]. Interestingly, inoculating these plants with the *Azospirillum* strain completely reversed this effect.

The role of ABA in plant defense responses to abiotic stresses, particularly under osmotic conditions such as high salinity and drought, is well documented. ABA induces short-term responses like stomatal closure, which aids in water balance regulation [27,128,129], and long-term growth responses via the regulation of stress-responsive genes. ABA plays a crucial role in the process of stomatal closure. An example of this is its regulation of the activation of the aquaporin gene TaAQP7, which is involved in water transport, under drought conditions [130].

Bacteria are assumed to have more than one pathway for ABA catabolism. For example, ABA was shown to be degraded by bacteria to phaseic and dihydrophaseic acids [121]. The soil bacterium Corynebacterium decomposes ABA to form a compound with similar spectral characteristics to dehydrovomifoliol [131].

### 2.6. Volatile Organic Compounds

Recent studies have revealed that volatile organic compounds (VOCs), secreted by a diverse array of soil bacteria, play a significant role in the stimulation of plant growth [16,132,133,134]. The increase in growth resulting from VOCs can be primarily related to the regulation of synthesis and metabolism of phytohormones produced by plants or PGPB [135,136]. The synthesis of VOCs by bacteria has been proven to have a plant-specific and compound-dependent effect, leading to an increase in plant photosynthesis and the modulation of gibberellin, auxin, and cytokinin levels. Furthermore, there have been reports indicating that VOCs can decrease ethylene levels in plants and impede the growth of certain fungal diseases. For example, endophytic strains of *Serendipita* that produce VOCs were found to be able to increase the yield and biomass of Arabidopsis seedlings in in vitro experiments. The mixture of VOCs produced by the bacteria rather than the individual VOCs was reported to affect several growth parameters of Arabidopsis plants including petiole elongation, expansion of lateral root epidermal cells, increased leaf area, increased maximum quantum efficiency of photosystem II (Fv/Fm), and increased anthocyanin accumulation [137].

There are volatile substances with unique effects on stimulating specific plant tissues or organs. These include 2,3-butanediol or acetoin, known to stimulate shoot biomass growth [138], and dimethylhexadecylamine, which exhibits antifungal effects [139].

According to the mVOC database, there are about 650 PGPB species that synthesize VOCs [140]. For example, VOCs produced by *Pseudomonas pseudoalcaligenes* (2-pentylfuran, dimethyldisulfide, and 2,3-butanediol) were shown to enhance maize plant growth and alleviated drought stress symptoms [141], and those produced by *Azospirillum brasilense* were shown to stimulate maize growth.

Studies have indicated that the release of VOCs by PGPB can effectively hinder potential pathogens. For example, the production of antifungal volatiles, including 2-methylfuran, benzene, 2-methyl-1-butanol, and myrcene, has been observed in *Streptomyces rochei* [142]. The strains *Pseudomonas koreensis*, *P. fluorescens*, *Lysinibacillus sphaericus*, and *Paenibacillus alvei* are known for their ability to produce a wide range of VOCs including acids, alcohols, alkanes, alkenes, aldehydes, amines, furans, ketones, pyrazines, salicylic acid sulfides, and terpenoids. It should be noted that the production of some of these compounds is specific to certain species or strains [143]. A published database of identified mVOCs (MVOC database 2.0, http://bioinformatics.charite.de/mvoc/) contains more than two thousand compounds produced by nearly one thousand microbial species [140,144]. Bacterial volatiles typically consist of alkenes, alcohols, ketones, terpenes, benzenoids, pyrazines, acids, and esters. For example, *Bacillus megaterium* produces heneicosane, heptacosane, and octacosane [145], *Paenibacillus polymyxa* produces 2-nonanone and 3-hydroxy-2-butanone [146], *Serratia plymuthica* produces dimethyltrisulphide and β-phenylethanol [147], and *Pseudomonas stutzeri* produces dimethyldisulphide [148]. All these substances possess antifungal activity.

### 2.7. Ethylene and ACC Deaminase

Ethylene, the earliest discovered gaseous plant hormone, is a key regulator of plant growth and development. In 1965, ethylene was recognized as a plant hormone [149]. The gaseous nature of ethylene facilitates its diffusion into nearby cells, resulting in ethylene production predominantly occurring locally at the site of its action [150]. Ethylene plays a crucial role in regulating various aspects of plant growth, including root formation, seed germination, fruit ripening, flower wilting, leaf fall, stress signaling, and the biosynthesis of various phytohormones [151,152]. There is a tendency for plants to generate limited amounts of ethylene, with a positive impact on their growth and development. Besides regulating plant growth and development, ethylene is involved in regulating plant responses to various biotic [153,154] and abiotic stresses [155,156]. A significant elevation in the endogenous biosynthesis of ethylene, known as “stress ethylene”, is often observed as a result of various stresses [157]. The positive role of ethylene as a mediator in promoting salinity stress tolerance in plants has been highlighted [158,159].

**Figure 6:**
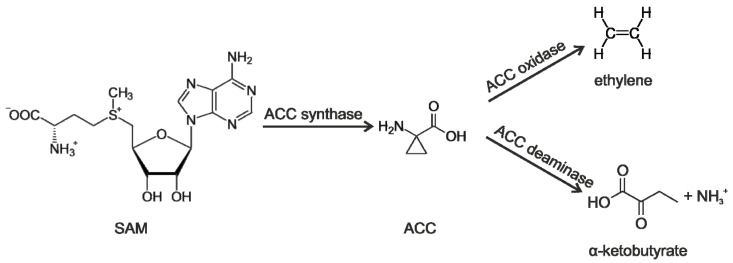
Pathway of ethylene biosynthesis and regulation of its concentration under the action of ACC deaminase enzyme. SAM—S-adenosylmethionine; ACC—1-aminocyclopropane-1-carboxylic acid.

The biosynthesis of ethylene in plants commences with the conversion of S-adenosylmethionine into ACC, facilitated by the enzyme ACC synthase. This is followed by the conversion of ACC into ethylene, catalyzed by the enzyme ACC oxidase [66]. Specific bacterial strains, such as *Mesorhizobium* [160,161], *Rhizobium leguminosarum* [162], *Burkholderia phytophirmans* [163], *Pseudomonas fluorescens* [164], *Pseudomonas migulae* [165], and other strains, can effectively reduce the concentration of ethylene by secreting the enzyme ACC deaminase [166]. The enzyme facilitates the conversion of ACC to α-ketobutyrate and ammonia, leading to a decrease in ethylene levels in plants (Figure 6) [166].

To date, a multitude of ACC deaminases have been characterized. These are multimeric enzymes localized in the cytoplasm and using pyridoxal phosphate as a tightly bound cofactor, with subunit masses ranging from 35 to 42 kDa and native sizes from 100 to 112 kDa [167,168,169].

ACC desaminase-producing *Bacillus filamentosus*, *Janibacter indicus*, and *Brevibacterium casei* isolated from the rhizosphere of *Zygophyllum coccineum* were reported to mitigate the negative effects of salinity on wheat [170]. Under biotic and abiotic stress, ACC deaminase acts as one of the key enzymes by which PGPB can affect the development and growth of plants [171]. For example, bacterial strains derived from the rhizosphere, endosphere, and phyllosphere of plants residing in Antarctica demonstrate both cold tolerance and the capacity to synthesize ACC deaminases. It is of interest that isolates have been categorized as “cold-resistant and hyper-ACC-degrading bacteria”, for example, those from *Pseudomonas*, *Serratia*, and *Staphylococcus genera* [172].

Waterlogged soils, in which rice is commonly grown, are also categorized as abiotic stress conditions that can cause increased levels of ethylene. The inoculation of *Paenibacillus* and *Methylophaga* PGPB-producing ACC deaminase was found to stimulate rice growth [173]. It is a wild-type *Pseudomonas putida* strain, but not a mutant that cannot produce ACC deaminase, that stimulated the growth of canola plants under salt stress [174]. Similarly, a *Variovorax paradoxus* strain containing the ACC deaminase gene, but not the mutant, was demonstrated to improve the growth and yield of pea plants under drought conditions [175]. The expression of ACC deaminase has been reported to be a major modulator of plant growth in endophytic *Pseudomonas fluorescens* and *P. migulae*, with knockout forms not exhibiting such activity [152]. Hence, ACC produced and secreted by plants can attract the microbial strains that produce ACC deaminase in high concentrations [176].

It is worth noting that in certain instances, the ACC deaminase gene alone does not guarantee its synthesis. Seven strains out of the thirteen studied in [177] were found to contain the *acdS* gene, with two strains belonging to the genus *Mesorhizobium* being capable of producing the enzyme only during the symbiotic phase when localized inside the root nodule. It was later discovered that ACC deaminase genes in *Mesorhizobium* are governed by the promoter of the nitrogen fixation regulatory gene *nifA2* and are selectively expressed solely within root nodules [178].

Consequently, not only does the presence of ACC deaminase in bacterial strains provide plants with salt tolerance by decreasing ethylene synthesis, but it also enhances plant growth and development.

## 3. Synergistic Effects of PGPB on Plant Growth through the Interaction of Multiple Pathways

While PGPB can stimulate plant growth through various mechanisms, the literature lacks extensive exploration of the relationships among these mechanisms. Studies have confirmed the connection between ACC deaminase production and phosphate solubilization in PGPB, including *Bacillus*, *Burkholderia*, *Pseudomonas*, and *Variovorax*. ACC deaminase-expressing strains, for instance, have been shown to enhance nodule formation and growth in chickpeas while also stimulating phosphorus uptake [179].

### 3.1. Effect of IAA on ACC Deaminase and Ethylene Synthesis

**Figure 7:**
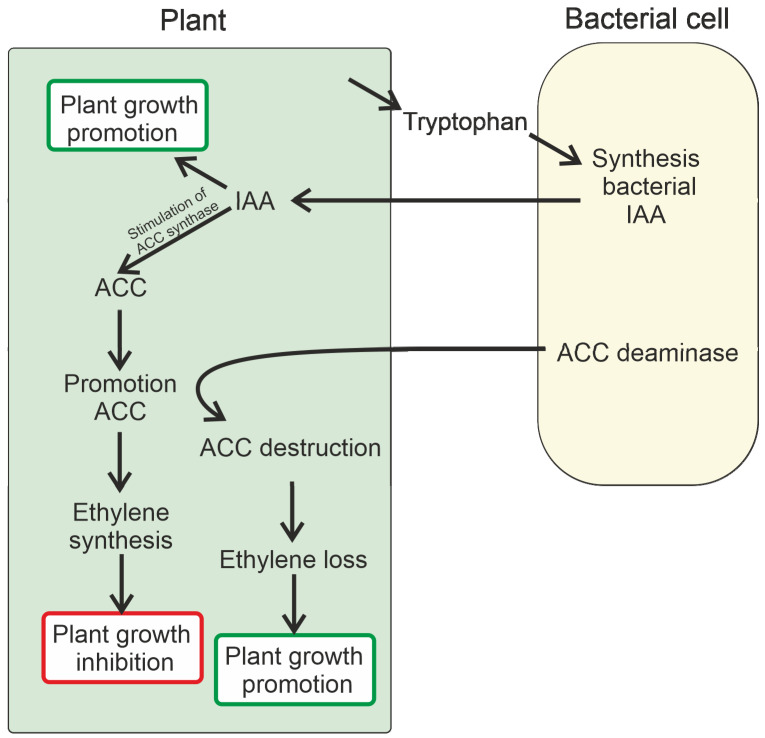
Schematic representation of the effect of IAA and ACC deaminase on plant growth.

PGPB can take up part of the tryptophan secreted by plants and convert it into IAA, which is secreted into the environment, and then IAA can be taken up by the plant [166]. IAA stimulates plant growth and activates the transcription of ACC synthase in the plant. As a result, the amount of ACC increases, which in turn leads to an increase in the concentration of ethylene in the plant. The presence of ACC deaminase in PGPB has a favorable effect on reducing the accumulation of newly synthesized ACC in plants, facilitated by the activity of the ACC deaminase enzyme. As a result, bacterial IAA can promote plant growth without causing substantial inhibition. In addition, low ethylene levels in a plant allow bacterial auxin to further stimulate plant growth [101]. This mechanism is schematically represented in Figure 7, with ACC eventually being converted into ammonia and α-ketobutyrate.

Almost all plant tissues and developmental stages are regulated by ethylene [101]. Ethylene synthesis in a given plant depends on the concentration of other phytohormones, temperature, gravity, light, nutrition, and the presence of different levels of biotic and/or abiotic stress to which the plant may be exposed [180]. The increase in ethylene concentration in plants is a response to various stresses [152,181]. One of the models describing the synthesis of “stress ethylene” includes ethylene synthesis in two peaks [182,183,184]. The first peak is associated with the consumption of the existing pool of ACC in stressed plant tissues [185]. The second, much larger ethylene peak occurs after the plant has synthesized additional ACC in response to stress. The second ethylene peak is generally detrimental to plant growth and is often associated with processes such as chlorosis and leaf drop. It is worth noting that the bacterial ACC deaminase possesses a remarkable ability to selectively reduce harmful levels of the second peak of ethylene while leaving the first smaller peak unaltered. The activation of plant defense responses is assumed to be dependent on the preservation of this first ethylene peak. ACC deaminase is normally present at relatively low levels in bacteria, with the induction of enzyme expression being a rather slow and complex process [157]. The induction of ACC oxidase in a plant (Figure 7) takes place during periods of stress, resulting in an initial small surge in ethylene production. Consequently, this triggers the transcription of defense genes in the plant. In addition, an increase in ACC concentration triggers the induction of bacterial ACC deaminase in response to the activation of ACC synthase in plants, leading to a secondary ethylene peak that is considerably diminished [166]. Given the higher affinity of ACC oxidase towards ACC compared with ACC deaminase, the levels of ethylene in the plant are contingent upon the ratio of ACC oxidase to ACC deaminase in the presence of ACC deaminase-producing bacteria [186]. In other words, to reduce ethylene levels successfully in plants, it is crucial for ACC deaminase to function prior to the significant induction of ACC oxidase.

Given that IAA activates ACC synthetase transcription, should we assume that the effect of IAA-producing bacteria on plants should result in the release of relatively high concentrations of ACC and, therefore, the inhibition of ethylene synthesis? This is actually not the case since the increase in ethylene levels in plants results in IAA synthesis inhibition, reducing the ACC synthase transcription [183,187,188]. PGPB that simultaneously secrete IAA and synthesize ACC deaminase do not increase ethylene levels in plants to the same extent as bacteria that secrete IAA but do not synthesize ACC deaminase [166]. The presence of ACC deaminase results in a substantial decrease in ethylene release, which inhibits IAA synthesis through a negative feedback mechanism. Thus, it is possible for bacterial IAA to continue to stimulate plant growth and increase ACC synthase expression, but most of the additional synthesized ACC will then be cleaved by bacterial ACC deaminase. In conclusion, the cross-reaction of IAA and ACC deaminase leads to the facilitation of plant growth stimulation by IAA, achieved through a reduction in ethylene levels by ACC deaminase (Figure 7).

### 3.2. Interactions among Phytohormones

Plants have a complex network of defense mechanisms that are activated upon infection by pathogens. SA, jasmonic acid (JA), ethylene, and ABA play a central role in this defense [189,190,191]. There is increasing evidence that these signaling pathways do not function independently but rather influence each other through a complex network of interactions [192]. When roots are colonized by certain microbes, infected by pathogens, attacked by herbivorous insects, or treated with chemicals, plants are able to respond by developing resistance. A distinction is made between systemic acquired resistance (SAR) [193,194] and induced systemic resistance (ISR) [195]. Colonization of roots by strains of non-pathogenic rhizobacteria leads to the development of ISR. This pathway primarily involves SA-independent signaling [196,197,198].

It has been shown that bacterial inoculation and the development of ISR results in increased expression of predominantly JA- and ethylene-regulated genes [199,200]. In the case of ISR, studies on different PGPB species and plants have shown that the type of resistance induced is, in most cases, independent of SA [201,202,203] and is generally associated with JA (and its derivatives, e.g., jasmonate) and ethylene [35,204]. PGPB were demonstrated to activate resistance against a wide range of pathogens through SA- or JA-dependent signaling pathways [205].

## 4. Strategies for Assessing the Ability of PGPB to Synthesize Phytohormones

Currently, various approaches are employed to examine the influence of PGPB on the regulation of plant hormones. One possible strategy is to discern the phytohormones synthesized by bacteria or substances that exert an impact on phytohormone production in plants. To provide an illustration, we consider the techniques for analyzing IAA production (Section 4.1) and ACC deaminase production by bacteria (Section 4.2).

The second strategy entails the identification of individual genes responsible for the production of phytohormones. For example, one method to detect PGPB with ACC deaminase activity is to search for genes, such as the *acdS* gene. The presence of the *acdS* has been documented in several endo- and epiphytic bacteria, including members of *Pseudomonas* [206,207]. An analysis conducted on over 200 strains revealed that nitrogen-fixing rhizobia was the predominant plant growth-promoting bacteria with ACC deaminase activity [208]. The screening of *acdS* genes indicated a significant level of diversity among rhizosphere bacteria of the *Poaceae* family [209] and other plant species [210]. Screening of *acdS* genes is used by some researchers for the initial selection of bacteria for further studies [211,212,213,214,215].

The third strategy involves generating mutant strains exhibiting phytohormone deficiencies or overproduction, followed by investigating the impact of these PGPB mutant strains on plant growth and development in comparison to the wild-type strain. Section 1 delves into a discussion of specific examples.

### 4.1. Determination of the Potential for IAA Synthesis

Various techniques have been employed to determine indolyl-3-acetic acid, including the use of gas–liquid chromatography with mass-selective detection [216,217]. The implementation of this approach necessitates costly apparatuses and intricate sample preparation.

### 4.2. Detection of ACC Deaminase Activity

PGPB that possess the ability to produce ACC deaminase are regarded as promising candidates for the purposes of inoculation and stimulation of plant growth. There are multiple approaches to the screening of PGPB with ACC deaminase.

The determination of PGPB with deaminase activity involves screening soil bacteria for their capacity to utilize ACC as the exclusive nitrogen source, indicative of the presence of the ACC deaminase enzyme. The isolates are inoculated and cultivated sequentially on PAF and DF (Dworkin and Foster) media [13]. One liter of PAF medium contains 10 g of proteose–peptone, 10 g of casein hydrolysate, 1.5 g of anhydrous MgSO_4_, 1.5 g of K_2_HPO_4,_ and 10 mL of glycerol [13]. One liter of DF minimal medium contains 4.0 g of KH_2_PO_4_, 6.0 g of Na_2_HPO_4_, 0.2 g of MgSO_4_ × 7H_2_O, 2.0 g of glucose, 2.0 g of gluconic acid, and 2.0 g of citric acid with trace elements including 1 mg of FeSO_4_ × 7H_2_O, 10 μg of H_3_BO_3_, 11.19 μg of MnSO_4_, 124.6 μg of ZnSO_4_ × 7H_2_O, 78.22 μg of CuSO_4_ × 5H_2_O, 10 μg of MoO_3_, pH 7.2, and 2.0 g of (NH_4_)_2_SO_4_ [218]. The deaminase activity of ACC can be assessed by quantifying the amount of α-ketobutyrate produced as a result of ACC deamination [13,219]. Section 4 discusses the screening of genes that exhibit deaminase activity.

## 5. Conclusions

Researchers have dedicated significant efforts to understanding how plants handle stress over the past few decades. It has become evident that plants, on their own, may not possess the mechanisms to combat abiotic stress effectively. The presence of beneficial soil bacteria is essential for their overall health and thriving, as they depend heavily on symbiotic relationships with them. A comprehensive understanding of how PGPB stimulate plant growth in unfavorable conditions is vital for effectively using bacteria as biofertilizers in agriculture. Diverse methodologies are used in the study of PGPB, including the detection of specific substances or hormones, comparing mutant and non-mutant forms of bacterial strains, screening individual genes, and using “omics” techniques. All these approaches contribute to our understanding of the interactions between PGPB and plants.

Maximizing the potential of PGPB–plant interactions in global crop production requires a detailed characterization of these interactions, with special attention to the phytohormones secreted and metabolized by the bacteria. The objective of this review was to examine the influence of PGPB on plant growth and development, with a particular emphasis on their role in hormonal regulation. PGPB can endogenously synthesize phytohormones and produce substances that regulate plant phytohormone levels. Thus, PGPB play an essential role in the hormonal regulation of plant life, particularly in challenging environmental conditions.

Despite the long-standing use of organic fertilizers and the introduction of mineral fertilizers in the 18th and 19th centuries, there is still much to be done to establish microbial fertilizers as an integral component of agricultural, horticultural, and silvicultural practices. Further detailed studies that cover various aspects are necessary to advance our understanding of plant–PGPB interactions.

## Data Availability

Not applicable.
